# Cilostazol protects against changes caused by streptozotocin-induced diabetic retinopathy

**DOI:** 10.5935/0004-2749.2021-0328

**Published:** 2022-07-04

**Authors:** Diego José Schebelski, Leandro Lipinski, Carlos Augusto Moreira Junior, Adriana Yuriko Koga, Bruna Carletto, Mario M. Montemor, Paulo Vitor Farago, Ricardo Zanetti Gomes

**Affiliations:** 1 Universidade Estadual de Ponta Grossa, Ponta Grossa, PR, Brazil; 2 Universidade Federal do Paraná, Curitiba, PR, Brazil

**Keywords:** Cilostazol, Diabetic retinopathy, Ganglion cell, Retina, Rats, Wistar, Cilostazol, Células ganglionares, Retinopatia diabética, Retina, Ratos Wistar

## Abstract

**Purpose:**

This study investigates the protective effect of cilostazol on the development and evolution of diabetic retinopathy in rats.

**Methods:**

Sixty male rats were divided into four groups: untreated nondiabetic rats, untreated diabetic rats, cilostazol-treated nondiabetic rats, and cilostazol-treated diabetic rats. The thickness of the internal limiting membrane to the outer limiting membrane, inner plexiform layer, inner nuclear layer, and outer nuclear layer were measured. The number of cell nuclei per 50-µm length in retinal sections was counted to quantify the degree of retinal cell loss.

**Results:**

The number of nuclei in the ganglion cell layer was significantly higher in untreated nondiabetic rats (p<0.05). The mean number of nuclei in the cilostazol-treated nondiabetic rats was significantly higher than that in the cilostazol-treated diabetic rats (p<0.05). The cilostazol-treated nondiabetic rats had a significantly higher mean nuclei count in the inner nuclear layer and inner plexiform layer as compared with the other groups (p<0.05). The total mean retinal thickness of the cilostazol-treated nondiabetic rats was significantly higher than that of cilostazol-treated diabetic rats and untreated diabetic rats (p<0.05).

**Conclusion:**

By decreasing the loss of ganglion cells and reducing the sensorineural atrophy in the internal retinal layers, cilostazol had a protective effect against changes caused by diabetic retinopathy in diabetic rats.

## Abstract (pt)

**Objetivo:**

O objetivo deste estudo foi investigar o efeito protetor do cilostazol no desenvolvimento e na evolução da retinopatia diabética em ratos.

**Métodos:**

Sessenta ratos machos foram divididos em 4 grupos: ratos não-diabéticos não-tratados, ratos diabéticos não-tratados, ratos não-diabéticos tratados com cilostazol e ratos diabéticos tratados com cilostazol. A espessura da membrana limitante interna à membrana limitante externa, a camada plexiforme interna, a camada nuclear interna e a camada nuclear externa foram medidas. Para quantificar o grau de perda de células da retina, foi contado o número de núcleos de células por 50 µm de comprimento em secções retinianas.

**Resultados:**

O número de núcleos no GCL foi significativamente maior em Ratos não-diabéticos não-tratados com cilostazol (p<0,05). O número médio de núcleos em Ratos não-diabéticos tratados com cilostazol foi significativamente maior do que em Ratos diabéticos tratados com cilostazol (p<0,05). A contagem média de núcleos em camada nuclear interna e camada plexiforme interna de ratos não-diabéticos tratados com cilostazol foi significativamente maior do que nos outros grupos (p<0,05). A espessura retiniana média total de Ratos não-diabéticos tratados com cilostazol foi significativamente maior do que em Ratos diabéticos tratados com cilostazol e Ratos diabéticos não-tratados (p<0,05).

**Conclusão:**

Os resultados demonstraram que o cilostazol teve um efeito protetor contra as alterações causadas pela retinopatia diabética em ratos diabéticos, diminuindo a perda de células ganglionares e reduzindo a atrofia neurossensorial nas camadas retinianas internas.

## INTRODUCTION

Diabetic retinopathy (DR) is one of the most common causes of irreversible blindness worldwide and the main reason for visual loss among people of working age. Individuals with more than 10 years of poor glycemic control have a greater chance of developing DR^(1)^. A previous study estimated that after 15 years of illness, 80% of patients with type 2 diabetes mellitus (DM) and 97% of those with type 1 DM will experience some degree of retinopathy^(2)^.

DR is a neurovascular complication specific to diabetes and has a significant effect on health systems around the world^(3)^. In 2010, this complication affected more than 100 million worldwide patients, and this number will continue to increase, affecting more than 190 million people by 2030^(4)^. The number of individuals with visual impairment resulting from DR and the proportion of individuals with blindness are increasing remarkably^(4)^.

Patients with diabetes develop a prothrombotic state that includes endothelial dysfunction, platelet dysfunction, and impaired coagulation^(5-7)^. DR is characterized by capillary closure and retinal ischemia, followed by the growth of new vessels in the retina and optic nerve. Visual loss can result from bleeding in the vitreous and retinal detachment secondary to vitreous-retinal traction^(8)^.

As compared with individuals without diabetes, patients with DR experience a reduction in retinal blood flow^(9)^. Nonperfusion of the deep capillaries is associated with a disruption of photoreceptors in ischemic diabetic maculopathy^(10)^. A proinflammatory state can arise due to the disturbance in the antioxidant system caused by hyperglycemia^(11)^. Many studies performed in patients with diabetes and in models of diabetic animals have indicated that tissue hypoxia and immune deregulation can lead to the progressive expression of intravitreal inflammatory molecules, including cytokines, chemokines, and other inflammatory factors responsible for DR development^(12-14)^. Evidence has shown that neuroinflammation and neurodegeneration play significant roles in the pathophysiology of early DR. Improving microcirculation in the retina and choroid, if associated with neuroprotection, could be an important therapeutic target^(15)^.

Cilostazol is a selective inhibitor of phosphodiesterase III that has antiplatelet, antithrombotic, and vasodilatory properties. Previous studies have suggested that the vasodilation induced by cilostazol may depend on nitric oxide (NO) from the endothelium^(16)^. The vasodilating effect of cilostazol has reduces hypoxia and ischemia. In addition, its anti-inflammatory properties also reduce the excessive production of blood vessels, which decreases vascular endothelial growth factor (VEGF) expression^(15)^. Treatment with cilostazol can culminate in the protection of retinal ganglion cells via reduced ischemia caused by diabetes, as reflected by a reduction in the release of VEGF^(17)^.

Taking into consideration the pharmacologic potential of cilostazol on DR, this study aims to evaluate the protective effect of cilostazol on the development and the evolution of DR in diabetic rats.

## METHODS

### Animals

We used 60 male, 90-day-old Wistar rats (*Rattus norvergicus*) from the Central Animal Facility of the State University of Ponta Grossa. The rats had a mean weight of 335 g (standard deviation = 44.6 g). All animals were kept in cages, with a maximum of five animals per cage. Water and commercial feed were provided ad libitum in an environment with controlled temperature and humidity. The study was previously approved by the Ethics Committee on the Use of Animals (No. CEUA - 017/2018).

### Diabetic rat model

The rats were fasted for 4 hours. Capillary glycemia was measured in the animals’ tails after weighing. Subsequently, we administered 40 mg/kg of streptozotocin (40 g/l) diluted in saline via peritoneal injection. On the second day, the animals’ blood glucose levels were measured again. Animals with blood glucose level <150 mg/dl received a new injection of 40 mg/kg of streptozotocin, and those with a blood glucose level between 150 and 250 mg/dl received a new injection of 20 mg/kg of streptozotocin. In rats with glycemia >250 mg/dl, no further administration of streptozotocin was performed. On the seventh day of the experiment, a new measurement of the capillary glycemia was obtained to prove the establishment of the diabetic rat model (capillary glycemia >200 mg/dl). We excluded animals that did not develop DM from the experiment. Control rats received an intraperitoneal injection of saline solution alone to ensure a proper match with the streptozotocin groups.

### Cilostazol administration

We randomized the animals and divided them into four groups of 15 animals each (Table 1). In the groups receiving cilostazol, we administered oral cilostazol (Infinity Pharma, Campinas, Brazil) by gavage at a dose of 30 mg/kg during a period of 8 weeks. Other groups received propylene glycol and water (70:30, v/v) as vehicle for gavage.

**Table 1:** Groups and treatments^a^

**Group**	**Treatment**
Untreated nondiabetic rats	Oral vehicle
Untreated diabetic rats	Oral vehicle
Cilostazol-treated nondiabetic rats	Oral cilostazol (30 mg/kg)
Cilostazol-treated diabetic rats	Oral cilostazol (30 mg/kg)

a = Oral treatment was performed in all animal groups.

### Tissue removal

All rats were euthanized after 8 weeks of treatment using an intraperitoneal overdose of general anesthesia (xylazine and ketamine). We checked the animals’ weight and capillary glycemia (Table 2). The eyes were enucleated and divided in half on the horizontal meridian, including the optic nerve, and then fixed in 10% formalin for later inclusion into paraffin.

**Table 2:** Mean ± standard deviation of weight and capillary glycemia on euthanasia day

**Group**	**Weight (g)**	**Glycemia (mg/dL)**
Untreated nondiabetic rats	372.0 ± 52	85.6 ± 9.2
Untreated diabetic rats	326.9 ± 45	269.9 ± 128.5
Cilostazol-treated nondiabetic rats	373.9 ± 38	96.5 ± 8.6
Cilostazol-treated diabetic rats	296.0 ± 46	330.4 ± 130.1

### Histological analysis of the retina

For histological analysis, the blocks were sectioned along the horizontal meridian with sections perpendicular to the retina. Hematoxylin and eosin staining was used. We discarded oblique sections, artifacts from the technique, which resulted in a thicker retina.

### Thickness of the retina layers

We performed morphometric analysis of the retina in accordance with the histological analysis model^(18)^. We prepared and analyzed histological images using the default tools of ImageJ software^(19)^. The thickness of four different retinal layers was measured: the thickness of the internal limiting membrane to the outer limiting membrane, inner plexiform layer, inner nuclear layer (INL), and the outer nuclear layer.

### Retinal cell count

Using ImageJ software, we counted the number of cell nuclei per 50-µm length in the retinal sections with linear cell densities to quantify the degree of retinal cell loss. We counted the number of cell nuclei of three retinal layers (ganglion cell layer [GCL], INL, and ONL) in a 50-µm width in the retina of both hemispheric sections at a distance of 1.5 mm from the head of the optic nerve.

### Statistical analysis

We initially performed a descriptive analysis of the data. Then, we assessed the difference between the groups using analysis of variance, followed by Tukey’s test as a post hoc measure (parametric approach for normal distributions) or Kruskal-Wallis test followed by Dunn’s test as a post hoc measure (nonparametric approach for nonnormal distribution). The tests were considered significant when p<0.05, and we performed all analyses using R software (R Core Team).

## RESULTS

### Histological analysis of the retina

**Figure 1:**
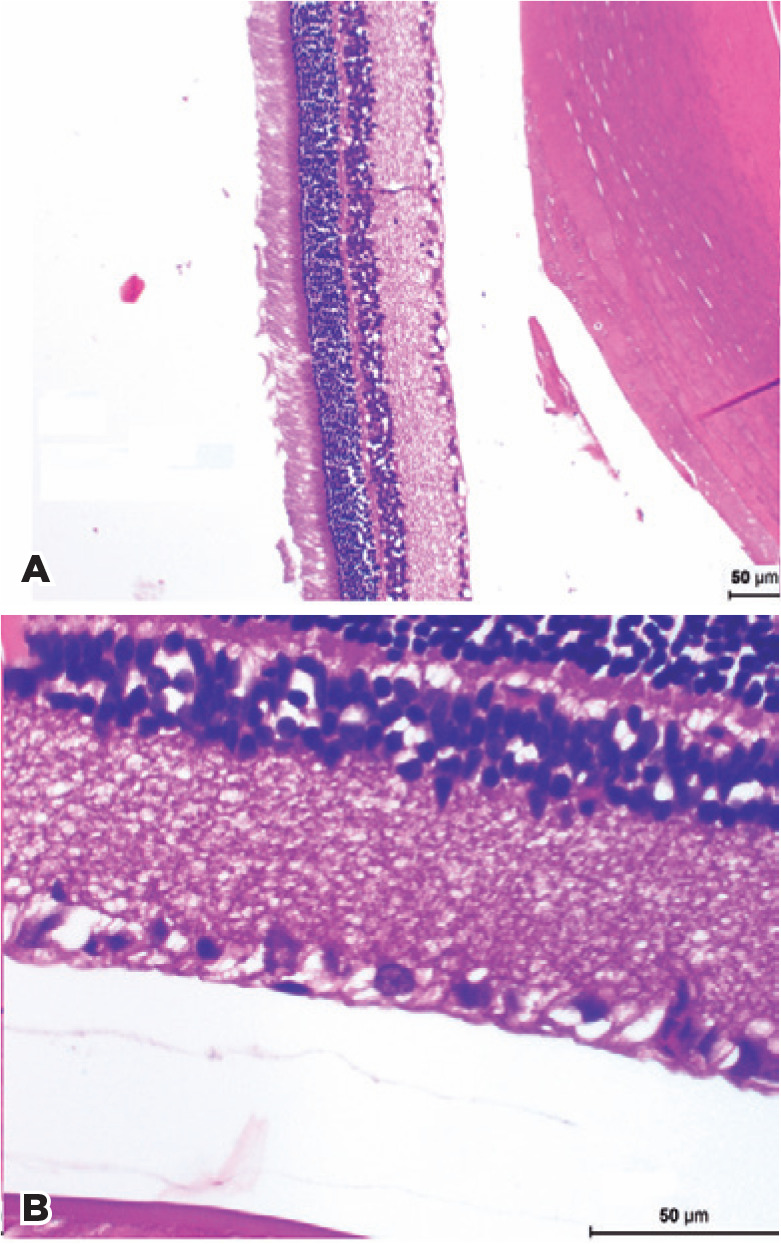
Representative histological images for cilostazol-treated diabetic rats. (A) Total retinal thickness, 100× magnification. (B) Ganglion cell layer, 400× magnification.

Figure 1 displays representative histological images of the total retinal thickness and the GCL for cilosta­zol-treated diabetic rats. From these micrographs, it can be seen that the histological sections showed suitable quality for the quantitative analyses.

### Thickness of the retinal layers

Table 3 describes the results of the morphometric analysis of the retina for the animal groups. Table 4 summarizes the *p* statistical data for the groups of rats.

**Table 3:** Mean (M) and standard deviation (SD) from the retinal morphometric analysis

	UN		CN		UD		CD		p value
	M	SD	M	SD	M	SD	M	SD	
Ganglion cells	4	1	5	1	3	1	4	0	<0.001
Inner nuclear layer (cells)	35	3	44	7	35	5	36	6	0.001
Outer nuclear layer (cells)	122	16	131	24	111	14	116	13	0.100
Retina thickness (mm)	0.179	0.028	0.190	0.021	0.174	0.013	0.165	0.015	0.021
Inner plexiform									
Layer thickness (mm)	0.069	0.010	0.078	0.008	0.071	0.006	0.069	0.003	0.006
Inner nuclear layer thickness (mm)	0.029	0.004	0.032	0.005	0.030	0.003	0.027	0.006	0.154
Outer nuclear layer thickness (mm)	0.055	0.011	0.055	0.008	0.050	0.007	0.051	0.006	0.424

UN= untreated nondiabetic rats; CN= cilostazol-treated nondiabetic rats; UD= untreated diabetic rats; CD= cilostazol-treated diabetic rats.

**Table 4:** Statistical comparison for the morphometric analysis between paired groups (p-values)

	**Paired groups**					
	**UN** × **CN**	**UN** × **UD**	**UN** × **CD**	**CN** × **UD**	**CN** × **CD**	**UD** × **CD**
Ganglion cell count	0.270	<0.001	0.281	<0.001	0.033	<0.001
Inner nuclear layer	<0.001	0.867	0.610	0.001	0.008	0.566
Total retinal thickness	0.112	0.943	0.237	0.036	0.002	0.151
Internal plexiform layer	0.009	0.847	1.000	0.104	0.020	0.887

UN= untreated nondiabetic rats; CN= cilostazol-treated nondiabetic rats; UD= untreated diabetic rats; CD= cilostazol-treated diabetic rats.

Table 3 shows that the ganglion cell count, INL cell count, total retinal thickness, and thickness of the inner plexiform layer were statistically significantly difference among the groups. The cell count of the ONL, the INL thickness, and the ONL thickness were not significantly different among the groups.

Table 4 shows that in the ganglion cell count differed significantly from the following groups: untreated nondiabetic rats (UN) × untreated diabetic rats (UD), cilostazol-treated nondiabetic rats (CN) × UD, CN × CD, and UD × CD. The INL cell count was statistically significantly different between the following groups: UN × CN, CN × UD, and CN × CD. The measure of total retinal thickness was also significantly different between the CN × UD and CN × CD groups. Furthermore, the *p* value showed a statistically significant difference in the measure of the internal plexiform layer between the UN × CN and CN × CD groups.

### Ganglion cell count

**Figure 2:**
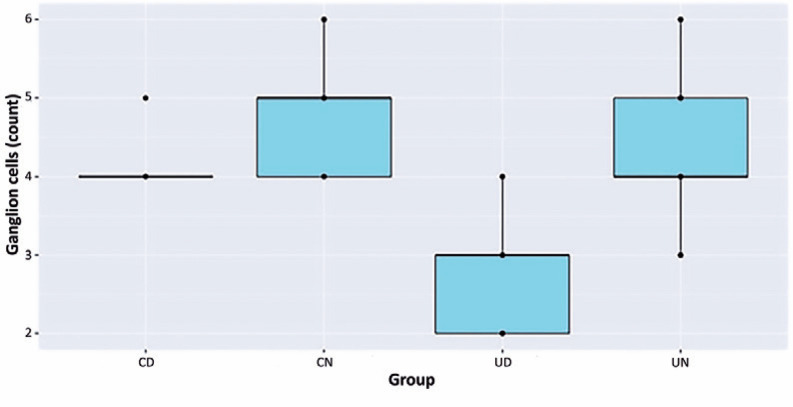
Ganglion cell count.

The number of nuclei in the GCL was significantly higher in the UN rats compared with the UD rats (p<0.05; Figure 2). The nucleus count in the CD rats was significantly higher than in the UD rats (p<0.05). In addition, the mean number of nuclei in the CN rats was significantly higher than in the CD rats (p<0.05). However, we found no statistically significant difference in the mean number of nuclei between UN rats and CN rats (p>0.05). Likewise, there was no statistically significant difference in the mean number of nuclei between the UN rats and CD rats (p>0.05).

* Distribution of ganglion cell count among groups.

UN= untreated nondiabetic rats; UD= untreated diabetic rats; CN= cilostazol-treated nondiabetic rats; CD= cilostazol-treated diabetic rats.

### Inner nuclear layer

The mean nuclei count in the INL of the CN rats was significantly higher than in the UD rats, CD rats, and UN rats (p<0.05). However, we found no statistically significant difference between UN rats and UD rats (p>0.05). Furthermore, no statistically significant difference was detected between CD rats and the UD rats (p>0.05).

### Total retinal thickness

We found that the mean total retinal thickness in the CN rats was significantly higher than in the CD rats and UD rats (p<0.05). However, no statistically significant difference was noted in the mean total retinal thickness of the CN rats compared with the UN rats (p>0.05). Likewise, we found no significant difference in the mean total retinal thickness between the CD rats and the UD rats (p>0.05).

### Inner plexiform layer thickness

The CN rats had a significantly higher internal plexiform layer thickness than the CD rats and the UN rats did (p<0.05). However, there was no statistically significant difference in the mean internal plexiform layer thickness in the UN rats compared with the UD rats (p>0.05). Likewise, we did not find a statistically significant difference in the mean thickness of the internal plexiform layer between the CD rats and the UD rats (p>0.05).

## DISCUSSION

Morphometric analysis of the retina conducted in this animal experiment revealed no statistically significant difference in the retinal thicknesses of the UD rats after 8 weeks as compared with the UN rats, which is in line with previous results reported in the literature^(20)^. However, we did observe a statistically significant difference in the total retinal thickness and the thickness of the internal plexiform layer in the CN rats as compared with the CD rats and UD rats, which suggests a neuroprotective effect of cilostazol on the retina. This finding confirms the hypothesis of previous studies^(21)^.

A significant loss was noted in the ganglion cells in rat groups with 8 weeks of DM induction using streptozotocin, as demonstrated in previous studies using this same diabetic rat model^(21)^. The CD rats showed a lower loss of ganglion cells as compared with UD rats. Furthermore, the UN rats and the CD rats were statistically similar, which suggests that this drug can potentially protect ganglion cells. This effect could be related to a decrease in inflammatory factors and vasodilation that reduces tissue ischemia related to DR^(22)^ as well as a reduction in oxidative stress that prevents DR progression^(20)^.

Our experiment also demonstrated no significant difference in the mean cell nuclei count for INL and ONL between diabetic and nondiabetic rats. Supposedly, more than 8 weeks is required to show a statistically significant difference in the loss of cell nuclei in such retina layers for rats with DR. Previous studies observed a significant reduction in the thickness of both INL and ONL in diabetic rat models at 10 to 12 weeks of diabetes induction^(23)^.

We also found that, as compared with the other groups, CN rats had a higher nuclei count in the INL. This result suggests that cilostazol has a neuroprotective effect in other layers of the retina in addition to the GCL, which might be related to a reduction in cell apoptosis^(13)^.

Although cilostazol has been described as a drug that acts on platelet aggregation, some studies have reported that it can inhibit platelet-endothelial and platelet-leukocyte cell interactions via its antioxidative stress^(24)^ and anti-inflammatory activity. Iwata et al.^(25)^ suggested that cilostazol contributes to neuroprotective effects via its inhibitory effect on postischemic leukocyte-endothelial cells.

In comparing the morphometric analysis between the groups, we found significant differences in the innermost retinal layers of the diabetic rats, an area that corresponds to vascularization from the central retinal artery. This finding demonstrates that this area is more susceptible to deregulation of normal physiology secondary to hyperglycemia. Cilostazol might be a possible therapeutic agent because of its effectiveness in increasing ocular blood flow in patients with DR, mainly attributed to its effect on modulating retrobulbar circulation^(26)^. As previously shown via the use of cilostazol-loaded nanoparticles^(27)^, the morphometric preservation of the retina may be related to the increased level of VEGF and the conservation of electrophysiological function in the retina of diabetic animals. Another experiment demonstrated that the administration of cilostazol could reduce the implicit times of full-field electroretinogram in patients with nonproliferative DR^(28)^.

This effect might be related to the preservation of endothelial NO release, which consequently prevents the development of vascular alterations. Cilostazol can act on the NO system through the inhibition of phosphodiesterase activity, modulation of the effects of adenosine, and potentiation of NO action^(29)^.

Cilostazol demonstrated a protective effect against changes caused by DR in diabetic rats. This effect could be due to a decreased loss of ganglion cells and reduced sensorineural atrophy in the internal retinal layers.
